# Effect of 10 kV/m Electric Field Therapy in a Pressure Injury Model in Rats: An Innovative Preliminary Report

**DOI:** 10.3390/bioengineering12020183

**Published:** 2025-02-14

**Authors:** Mustafa Soner Özcan, Halil Aşcı, Pınar Karabacak, Eyyüp Sabri Özden, Rümeysa Taner, Özlem Özmen, Muhammet Yusuf Tepebaşı, Selçuk Çömlekçi

**Affiliations:** 1 Department of Anesthesiology and Reanimation, Faculty of Medicine, Suleyman Demirel University, Isparta 32260, Turkey; drpinara@gmail.com (P.K.); dreyupsabri@gmail.com (E.S.Ö.); 2 Department of Pharmacology, Faculty of Medicine, Suleyman Demirel University, Isparta 32260, Turkey; halilasci@sdu.edu.tr; 3 Department of Bioengineering, Institute of Science, Suleyman Demirel University, Isparta 32260, Turkey; rumeysataner21@gmail.com (R.T.); scom56@gmail.com (S.Ç.); 4 Department of Pathology, Faculty of Veterinary Medicine, Burdur Mehmet Akif Ersoy University, Burdur 15030, Turkey; ozlemoz@mehmetakif.edu.tr; 5 Department of Genetics, Faculty of Medicine, Suleyman Demirel University, Isparta 32260, Turkey; muhammettepebasi@sdu.edu.tr; 6 Department of Electronics and Communication Engineering, Faculty of Engineering, Suleyman Demirel University, Isparta 32260, Turkey

**Keywords:** bed sore, decubitus ulcer, electrical field, pressure ulcer, wound healing

## Abstract

**Background:** Pressure injuries are still an important health problem worldwide, although many therapies have been applied to date. This study aimed to determine the optimal duration of external application of a 10 kV/m direct current (DC, static) electric field in a pressure injury model in rats. **Methods:** Twelve male Wistar–Albino rats were divided into three groups: Grade-1, Grade-2, and Grade-3. Two round magnets were placed 4 h daily for one day in Grade-1, two days in Grade-2, and three days in Grade-3. Following wound formation, one rat from each group was designated the control, while the other rats were exposed to a 10 kV/m electric field for 15, 30, or 60 min. **Results:** Histopathological improvements were observed after 15 and 30 min of application, whereas a sharp decrease in the gene expression of growth factors at 30 min revealed that 15 min of application was optimal overall. **Conclusions:** According to the results of this study, 15 min applications of an external 10 kV/m electric field are promising for providing satisfactory results in wound healing. Further studies should examine in greater detail the effects of electric fields on growth factors and the mechanisms underlying these responses.

## 1. Introduction

Pressure injury (PI) is localized damage to the skin and/or underlying tissue caused by pressure, shear, or a combination of both, and it is a long-standing global healthcare problem that can be observed in all settings and affects millions of people [1,2]. Although it can affect people of all ages and health conditions, it is more common in elderly, immobile, intensive care patients with comorbidities [1,3,4]. There are significant physical, psychological, social, and economic burdens on affected individuals, caregivers, and the health care system [1,4,5]. Therefore, timely and effective treatments are needed.

Pressure injury treatment has traditionally relied on methods such as negative pressure wound therapy (NPWT), pharmacological agents, advanced wound dressings, and surgery for wounds refractory to other care [6]. NPWT accelerates healing by promoting angiogenesis and reducing edema through mechanical suction [7,8]. Pharmacological approaches often involve the use of growth factors, such as epidermal growth factor (EGF) and vascular endothelial growth factor (VEGF), or systemic anti-inflammatory agents to enhance tissue repair [9]. Advanced wound dressings, including silver-impregnated or collagen-based materials, offer bioactive environments for wound healing [10]. However, these methods have limitations in terms of cost, complexity, and patient compliance.

The pathophysiology of PIs begins with ischemia in the skin and underlying tissues due to prolonged pressure, which prevents adequate oxygen and nutrient delivery to the tissues. When pressure is relieved, sudden blood flow (reperfusion) increases free radical production, contributing to oxidative stress and tissue damage. The ischemia and reperfusion processes trigger an inflammatory response, attracting macrophages and neutrophils to the damaged area and accelerating ulceration [1,11]. Wound healing is a complex process that involves four overlapping, continuous, and interacting phases: hemostasis, inflammation, proliferation, and remodeling. Effective wound healing relies on coordinated interactions among various cell types, as well as a range of growth factors, cytokines, and chemokines [3,12]. These interactions contribute to the stabilization of the tissue.

Endogenous electrical field (EF) stimulation is thought to play an important role in all phases of wound healing, especially by promoting the migration of epidermal cells [13,14,15,16]. On the other hand, electrical stimulation (ES) therapy has been shown to enhance wound healing by increasing cell proliferation through the upregulation of various growth factors and insulin receptors in fibroblasts, accelerating blood flow to the wound, exerting anti-inflammatory effects, and increasing intercellular conduction. ES also accelerates scar maturation and remodeling by increasing α smooth muscle activity and type 1 collagen production. It also improves circulation by stimulating the superficial skin vascular endothelium to release nitric oxide (NO), which dilates blood vessels [17,18,19]. Previous studies have shown that electric fields modulate these angiogenetic responses through the activation of both VEGFs and endothelial nitric oxide synthase (eNOS) in endothelial cells [20,21].

Currently, studies on the effects of exogenous EFs on wound healing, especially those applied without contact with the skin, are lacking [22]. Therefore, the use of electrical therapy without touching the patient is an innovative idea and is also cost-effective, easy to use, and accessible [18,19]. Unlike contact-based therapies, non-contact EF application reduces the risk of adverse effects such as skin irritation and pain, offering a cost-effective and accessible solution. The novelty of this study lies in its examination of a 10 kV/m electric field applied without skin contact, with the goal of identifying the optimal treatment duration for effective wound healing. Thus, an innovative preliminary study of the biological effects that we want to investigate in more detail will be completed.

## 2. Materials and Methods

### 2.1. Electric Field Application Setup

The exposure setup consists of a set of parallel plates with a power supply. The power supply is actually a power transformer. The AC output power transformer is rectified to obtain a high DC voltage. Since the EF density was E = 10 kV/m between the plates and the supply voltage was 8 kV, the desired electric field value was obtained for a plate spacing of 0.8 m. The reason for choosing to work with an electric field of 10 kV/m is that the World Health Organization’s exposure limit for the public in the main frequency supply voltages is 10 kV/m [https://apps.who.int/gho/data/view.main.EMFLIMITSPUBLICLOWv (accessed on 8 April 2023)].

A multimeter (Chauvin Arnoux Max 3000 TRMS, Paris, France) was used for voltage measurement. A digital Gauss/Tesla meter (Unilab, Blackburn, UK) was used to demonstrate the purity of the EF (magnetic field minimization). The maximum background noise of the AC and DC magnetic fields should be less than 1% (total unwanted field intensity of 0.001 mT). Thus, we can neglect the total magnetic field effects in the experimental environment. An electromagnetic field meter–industrial compatibility meter and its probes (HI-3804, Holaday Industries, Inc., Eden Praire, MN, USA) were used during all phases of the experiment.

**Figure 1:**
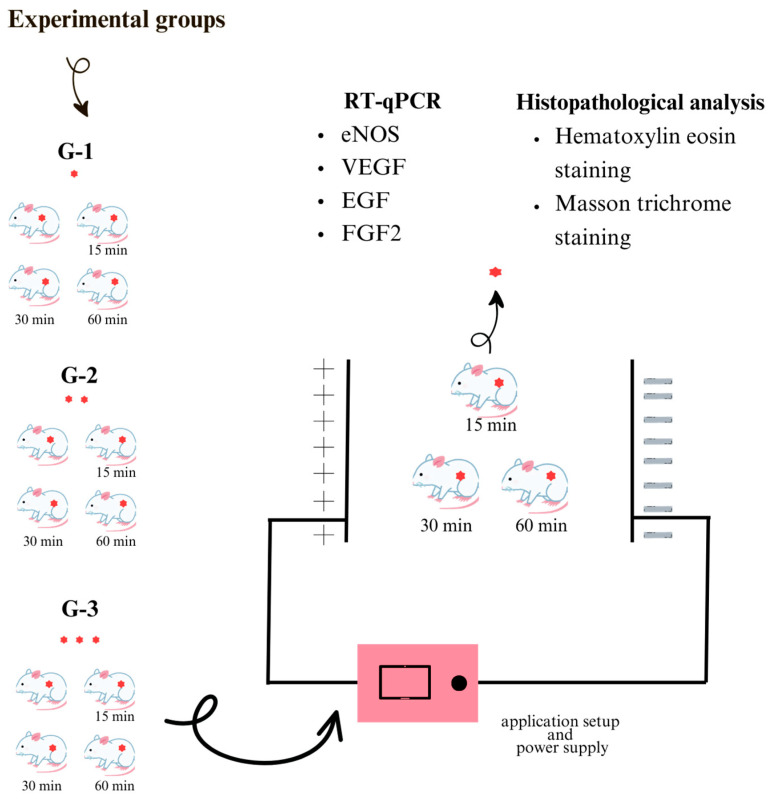
Experimental groups and 10 kV/m electric field (8 kV) high-voltage controller and parallel plate application setup. Red dots indicate pressure injuries.

To create EF exposure in the unit, two plates of 1.5 × 1 m dimensions were placed in parallel. The power supply was connected to the immersion galvanized sheet plates to create EF exposure, and the plates were isolated by using PTFE material isolation stands at the appropriate height to prevent contact with the ground. The purpose of the parallel plate experimental setup was to ensure that the animals in the cages were under the same value and homogeneous exposure, regardless of where they were in the cage. Cable connections were made in the center of each plate so that they did not physically cross the other side of the plate. The high-voltage control device is a control device capable of generating DC output. Inside the device, an on–off connection board was used to connect the main input to the protection circuit (Figure 1).

As shown in Figure 1, magnets are used to create pressure injuries in all animals before they are placed in the experimental setup, and the results of three different durations of application are examined.

### 2.2. Experimental Groups and Animals

A study was planned with the approval of the Suleyman Demirel University Animal Experiments Local Ethics Committee (SDU HADYEK) (Ethics No: 11.05.2023/05-171) for the purpose of the healing process with the application of a 10 kV/m electric field in the pressure injury model.

Twelve male Wistar–Albino rats weighing 300–350 g were obtained from the SDU Experimental Animals Laboratory. The rats were fed a normal diet and kept in special cages with 12 h of light and 12 h of darkness under room conditions at 22–24 °C and 55–60% humidity. All the rats were kept in the electric field unit for 0–1 h with the device inactive before the experiment started.

**Figure 2:**
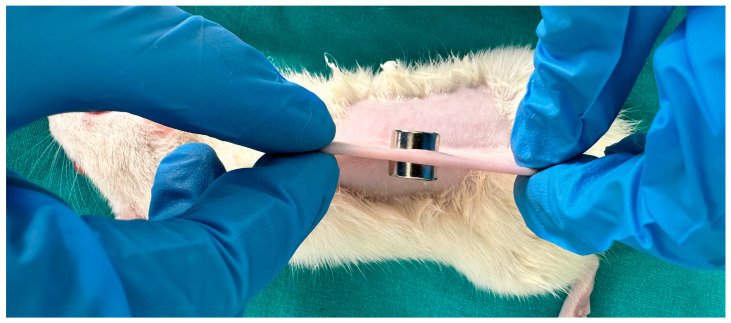
Pressure application to rat skin with magnets.

Considering both wound grade and application time separately, it was planned to use a minimum number of animals to determine the exact application time to be used, as an excessive number of animals would be required for the next planned experiment. Therefore, a specific statistical analysis could not be provided, and only histopathological and genetic analyses were planned. Accordingly, 12 rats were divided into three groups randomly: Grade-1 (G-1), Grade-2 (G-2), and Grade-3 (G-3). In all the groups, the rats were anesthetized with 90 mg/kg ketamine and 8–10 mg/kg xylazine, the back area was cleaned with 70% isopropanol, and the back hair was shaved. Stadler et al. created a pressure injury model in rat skin using 1000 Gauss mini-round magnets [23]. Taking this model as reference, the magnetic force value of the magnet we have (UNILAB Digital Gauss, Teslameter, UK) was measured as 2.69 mT. According to this measurement value, the time to be applied was determined as 4 h. In the G-1 group, the skin on the back was slightly pulled, and two round magnets (diameter: 12 mm and thickness: 5 mm) were placed for one day (4 h). In the G-2 and G-3 groups, round magnets were placed 4 h a day for two days and 4 h a day for three days, respectively. One rat from each group was used as the control, and the other rats were exposed to EF for 15–30–60 min (one rat for each period). The control group consisted of animals subjected to the same pressure injury induction process using magnets but not exposed to the electric field. This design was to ensure that any differences observed in the experimental groups could be attributed to the electric field treatment rather than the pressure injury model itself. The formation of a pressure injury with magnets is shown in Figure 2.

The same pressure application procedure was performed on each rat, as shown in Figure 2. Each group was exposed to 10 kV/m EF for the specified exposure duration. At the end of this process, the animals were anesthetized intraperitoneally with 80 mg/kg ketamine and 8–10 mg/kg xylazine and sacrificed. Half of the tissue samples were placed in 10% formaldehyde for hematoxylin–eosin staining for histopathological analysis of skin texture. The other halves were kept at −80 °C for genetic analysis of epidermal growth factor (EGF), vascular endothelial growth factor (VEGF), fibroblast growth factor 2 (FGF2), and eNOS gene expression (Figure 1).

### 2.3. Histopathological Analysis

At the end of the experiment, the wound area samples were collected and fixed in 10% neutral formalin during necropsy for histological examination. To prevent shrinkage at the defect site, the skin samples were secured on a flat surface, and formaldehyde was poured over the area containing the defect. After waiting for five minutes to harden, the samples were placed in a formalin solution. After 2 days of fixation, the skin samples were routinely processed by a fully automated tissue processor (ASP300S; Leica, Wetzlar, Germany). Following the paraffin wax embedding of the tissues, serial 5 µm thick sections were cut from the paraffin blocks using a rotary microtome (Leica RM2155; Leica, Wetzlar, Germany). The hematoxylin and eosin (HE) method was used to stain a series of sections for histopathological analysis, and the slides were coverslipped and examined via a light microscope.

The other serial sections were stained using a Trichrome Stain Kit (Connective Tissue Stain) (ab150686) for collagen (Abcam, Cambridge, UK) to evaluate connective tissue healing according to the manufacturer’s instructions and examined under a light microscope.

Microphotography and morphometric analyses were performed using the Database Manual Cell Sens Life Science Imaging Software System (Version 4.1) (Olympus Corporation, Tokyo, Japan).

Histopathological scores of reepithelization, neovascularization, granulation tissue, inflammatory cells, and ulcers were determined according to the criteria set in a previous study [24] and are presented in Table 1.

### 2.4. Reverse Transcription–Polymerase Chain Reaction (RT–qPCR)

Using the manufacturer’s protocol, RNA was isolated from homogenized tissues with the GeneAll RiboEx (TM) RNA Isolation Kit (GeneAll Biotechnology, Seoul, Republic of Korea). The amount and purity of the RNAs obtained were measured with a BioSpec-nano nanodrop (Shimadzu Ltd., Kyoto, Japan) device. One microgram of RNA was used for cDNA synthesis. cDNA synthesis was performed using an A.B.T.™ cDNA Synthesis Kit (Atlas Biotechnology, Ankara, Turkey) in a thermal cycler according to the manufacturer’s protocol. The primers were designed by detecting specific mRNA sequences and testing possible primer sequences via the NCBI website. The sequences of the primers used are shown in Table 2. The expression levels of genes were measured via a Bio-Rad CFX96 (Bio-Rad Laboratories, Inc., Hercules, CA, USA) real-time PCR instrument using the A.B.T.™ cDNA Synthesis Kit (Atlas Biotechnology, Ankara, Turkey). In this study, the GAPDH gene was used as a housekeeping gene. The reaction mixture was prepared according to the manufacturer’s protocol to a final volume of 20 µL. The resulting reaction mixture was placed in a real-time qPCR device with thermal cycling determined according to the kit manufacturer’s protocol, and each sample was studied in triplicate. The PCR conditions were as follows: initial denaturation at 95 °C for 300 s, 1 cycle of denaturation at 95 °C for 15 s, and annealing/extension at 54 °C for 30 s; 40 cycles were performed. Relative mRNA levels were calculated using the 2^−ΔΔCt^ method to normalize the results.

## 3. Results

### 3.1. Histopathological Findings

**Figure 3:**
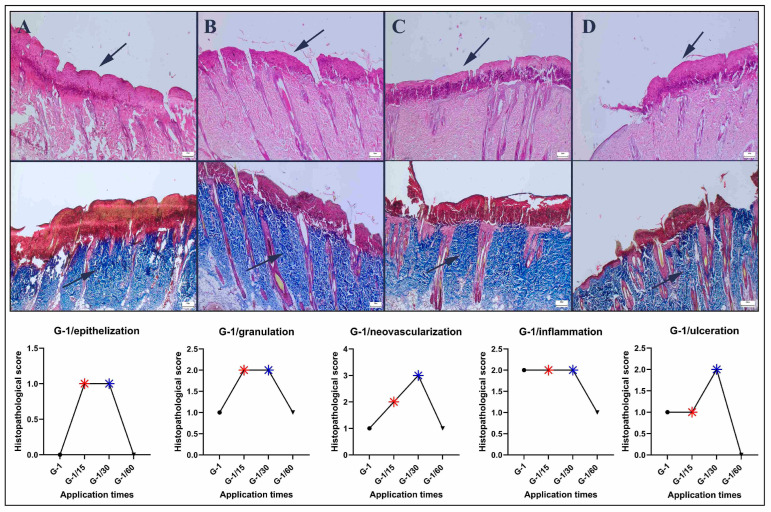
Histopathological findings of the Grade-1 group (upper row): (**A**) marked necrosis (arrow) in the control group; (**B**) decreased necrotic crust (arrow) in the 15 min group; (**C**) marked decrease in the necrotic area in the 30 min group; and (**D**) marked ulcer and necrotic area (arrow) in the 60 min group, HE staining. Collagen quality in the defect area (lower row): (**A**) irregular and mildly mature collagen (arrow) in the control group; (**B**) irregular and moderately mature collagen in the 15 min group; (**C**) regular and mature collagen (arrow) in the 30 min group; and (**D**) immature and irregular collagen (arrow) in the 60 min group, red areas indicate crust, while blue areas indicate collagen, Masson’s trichrome method, scale bars = 200 μm.

Histopathological examination at 4 h per day for one day revealed widespread necrosis and crust formation in the region where pressure injury was successfully induced in the control group. After the 15 min treatment, a noticeable reduction in necrosis and ulceration was observed. However, during the 30 min treatment, a significant improvement in the necrotic area compared with that during the 15 min was observed. However, the 60 min treatment increased necrosis and had destructive effects on all the examined criteria (epithelialization, neovascularization, granulation tissue, and inflammation) (Figure 3).

**Figure 4:**
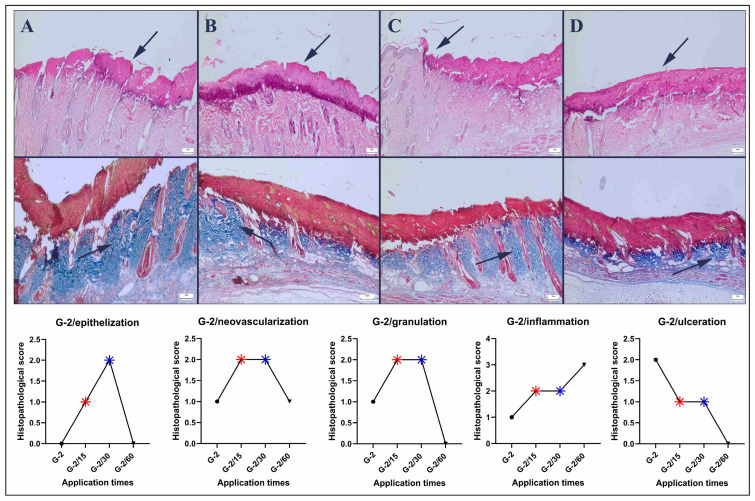
Histopathological findings of the Grade-2 group (upper row): (**A**) marked necrosis (arrow) in the pressure area of the control group; (**B**) reduced necrotic crust thickness (arrow) in the 15 min group; (**C**) significant decrease in the necrotic area in the 30 min group; and (**D**) pronounced ulcer and necrotic area (arrow) in the 60 min group, HE staining. Collagen quality in the defect area (lower row): (**A**) irregular and mildly mature collagen (arrow) in the control group; (**B**) irregular and moderately mature collagen in the 15 min group; (**C**) regular and mature collagen (arrow) in the 30 min group; and (**D**) immature and irregular collagen (arrow) in the 60 min group, red areas indicate crust, while blue areas indicate collagen, Masson’s trichrome method, scale bars = 200 μm.

When groups in which pressure injuries were induced for 4 h per day for two days with pressure application were examined, significant necrosis and crust formation were observed in the control group. In the 15 min treatment group, a reduction in pathological findings was noticeable, and in the 30 min treatment group, a significant improvement was evident. However, a 60 min application led to further exacerbation of the pathological findings (Figure 4).

**Figure 5:**
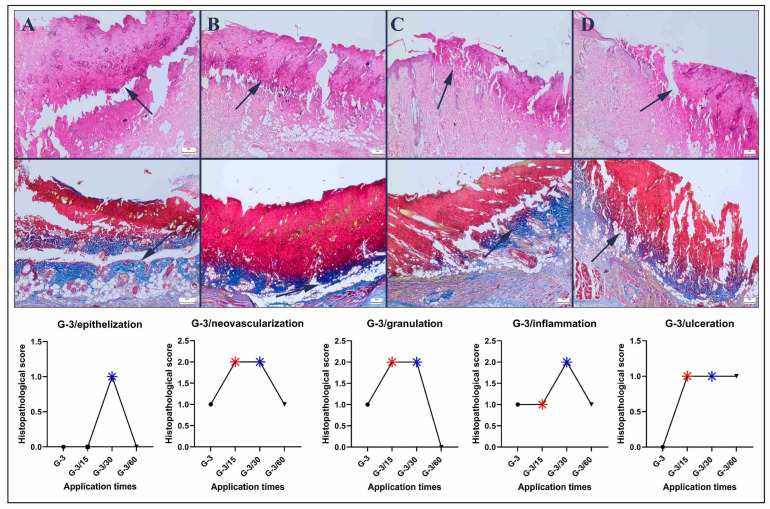
Histopathological findings of the Grade-3 group (upper row): (**A**) marked tissue loss and necrotic area (arrow) included subdermal tissue in the pressure area of the control group; (**B**) relatively reduced necrotic area (arrow) in the 15 min group; (**C**) marked decrease in the necrotic area in the 30 min group; and (**D**) marked ulcers and necrotic area that included the subdermal area (arrow) in the 60 min group, HE staining. Collagen quality in the defect area (lower row): (**A**) irregular, thin, and mildly mature collagen (arrow) in the control group; (**B**) irregular, thin, and moderately mature collagen in the 15 min group; (**C**) thick, regular, and mature collagen (arrow) in the 30 min group, and **(D**) very thin, immature and irregular collagen (arrow) in the 60 min group, red areas indicate crust, while blue areas indicate collagen, Masson trichrome method, scale bars = 200 μm.

Histopathological examination of the groups subjected to pressure injuries for 4 h per day for three days revealed severe tissue loss in all the groups, with noticeable disruption of tissue integrity in the control group. In the 15 min treatment group, a decrease in pathological findings was observed, whereas the 30 min treatment group presented more pronounced improvement. However, a 60 min application led to further intensification of the pathological findings (Figure 5).

The results of this study indicated that a single application lasting 4 h significantly induced a pressure injury model. The group that exhibited the most pronounced improvement underwent a 30 min treatment, whereas the 60 min treatment had a detrimental effect on the healing process. The 15 and 30 min treatments effectively promoted reepithelialization, neovascularization, and granulation tissue formation; reduced inflammation; and decreased the ulcer area.

### 3.2. RT–qPCR Results

**Figure 6:**
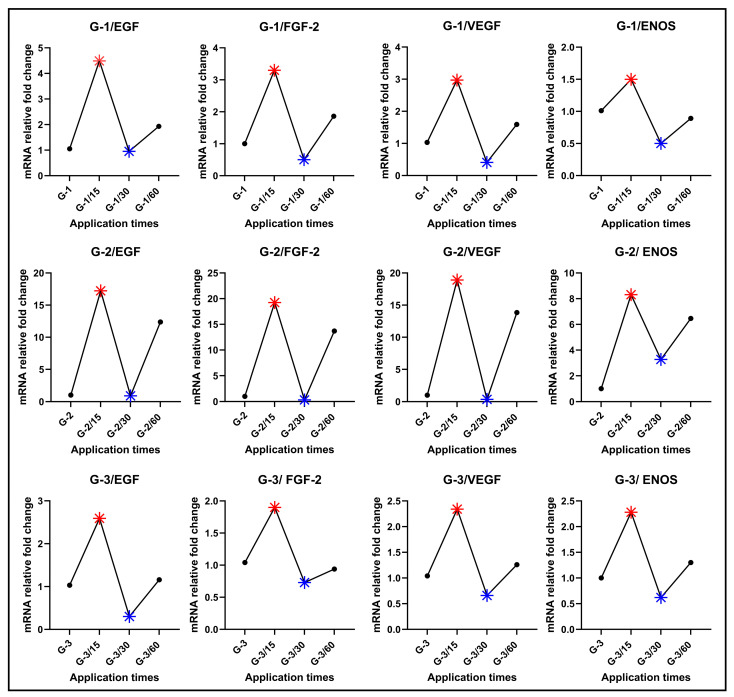
Relative fold changes in the mRNA expression of the EGF, FGF2, VEGF and ENOS genes with respect to skin texture. G-1: Grade-1; G-2: Grade-2; G-3: Grade-3; min: minute; RT–qPCR: reverse transcription–polymerase chain reaction; eNOS: endothelial nitric oxide synthase; VEGF: vascular endothelial growth factor; EGF: epidermal growth factor; and FGF2: fibroblast growth factor. Red areas indicate crust, while blue areas indicate collagen.

In the G-1 PI study groups, where 4 h pressure was applied once on the first day, and the G-3 PI study groups, where 4 h pressure was applied every day for three days, the expression levels of all EGF, FGF2, VEGF, and eNOS genes were determined at maximum levels in the 15 min application (4.5-fold, 3.5-fold, 3-fold, and 1.5-fold, respectively) (Figure 6). These expression levels subsequently sharply decreased at the 30th min and fell below the values of each grade without a device. The same markers sharply increased at the 15th min (1-fold, 0.5-fold, 0.3-fold, and 0.5-fold, respectively) and reached levels close to those of G-2 without devices at 30 min, except for eNOS. Therefore, the most suitable time for wound healing was the 15th min.

## 4. Discussion

This study presents significant findings regarding the effects of a 10 kV/m EF on a rat PI model, specifically detailing the relationships between histopathological parameters and the expression levels of growth factors (EGF, FGF2, VEGF, and eNOS) that are critical in wound healing. These markers play crucial roles in cell proliferation, angiogenesis, and tissue repair, offering insights into how their modulation over the treatment duration correlates with observed wound healing outcomes [20,21,25,26].

Histopathological examinations revealed a gradual improvement over the treatment duration with EF application. A 15 min application yielded the most marked improvements across all evaluated criteria (epithelialization, neovascularization, granulation tissue, and inflammation). Although the 30 min application also provided positive outcomes, its effect was comparatively limited. However, the 60 min application led to adverse effects such as extended necrosis and increased tissue damage, indicating that exceeding a critical treatment duration threshold might disrupt the healing process. Sari et al. reported better wound healing in terms of VEGF-positive cell density, reepithelialization, and collagen deposition in the 10 min ES group than in the other groups [27]. In their study, Sari et al. performed an application in which electrodes contacted the skin. However, since the mechanism of interaction at the cellular level is the same, we were able to make a comparison.

These findings align with the expression levels of growth factors. In both the G-1 (single 4 h pressure application) and G-3 (4 h pressure application per day for three days) groups, the 15 min treatment resulted in the greatest increase in the expression of growth factors such as EGF, FGF2, and VEGF. These molecular increases directly corresponded with the histopathological improvements observed, enhancing processes such as cellular migration, proliferation, and angiogenesis that expedite healing.

EGF accelerates the reepithelialization process by promoting the proliferation and movement of keratinocytes. EGF is also known to stimulate angiogenesis, myofibroblast activation, and the proliferation of epithelial cells and plays a role in closing the wound surface [28,29]. In this study, we found similar results for EGF, in which a similar situation was detected, indicating that 15 min of EF application increases epithelialization with increasing EGF. However, at 30 min, the expression levels of these markers sharply decreased, falling below those of the untreated groups. This suggests that while the EF initially elicits a strong healing response, prolonged exposure may adversely impact the regulation of these critical growth factors. Such overstimulation may trigger negative feedback mechanisms or cellular stress responses, resulting in decreased growth factor expression and worsened tissue pathology.

The expression levels of EGF, FGF2, VEGF, and eNOS support the conclusion that a 15 min treatment duration is optimal for wound healing. eNOS and VEGF contribute to the formation of new vessels by promoting angiogenesis, vasodilation, and the proliferation and migration of endothelial cells. FGF2 supports the differentiation and proliferation of endothelial cells in the neovascularization process. It also accelerates the formation of blood vessels by working together with VEGF [11,17]. In this study, vascularization of the wound area was achieved by increasing FGF2 and VEGF with 15 min of EF application.

These markers peaked during this timeframe, particularly in the G-1 and G-3 groups, and were directly associated with histological improvements. EGF and FGF2 support keratinocyte and fibroblast proliferation, promoting epithelialization and granulation tissue formation, whereas VEGF induces angiogenesis, facilitating oxygen and nutrient transport to the tissue. At each stage of wound healing, eNOS and NO production are fundamental processes that support cellular migration, proliferation, and tissue repair [30,31]. Therefore, regulating and optimizing eNOS activity can lead to faster and more effective wound healing. In particular, the desired increase in eNOS levels after 15 min of EF application may have contributed to the observed positive effects. Interestingly, in the G-2 PIs (two 4 h pressure applications), these markers, with the exception of eNOS, returned to values similar to those of the untreated groups after the 30 min treatments. These findings suggest that even a slight extension of the treatment duration may adversely impact growth factor production. Thus, EF therapy appears to be sensitive to duration, necessitating careful adjustment of the treatment time. A previous study revealed that eNOS activity increases over time in human umbilical vein endothelial cells exposed to a DC EF of 150 mV/mm; thus, NO production is induced [20]. In contrast, they reported a gradual increase in eNOS activity over time. This may be attributed to the difference in the EF densities applied.

These findings have significant implications for the clinical application of EF therapy in the treatment of PIs. The 15 min treatment duration, during which histopathological improvements and growth factor peaks align, may be the optimal timeframe for wound healing. However, the adverse effects observed with prolonged applications underscore the need for caution when extending treatment duration. Prolonged exposure to EFs may overstimulate cellular repair mechanisms, potentially triggering inflammatory or destructive responses. As emphasized by Katoh, prolonged electrical stimulation may result in hyperpolarization of the cell [25]. Hence, determining the optimal duration was crucial in this study.

In this study, the application of the treatment without placing electrodes on or around the wound can be considered advantageous because of the absence of potential adverse events such as skin irritation, muscle contraction, and pain, considering the studies performed to date [32]. 

This study has several limitations. First, since this is a preliminary study, the context and sample size of this study need to be expanded to make the results more generalizable. Second, since this study is an experimental model and was performed on healthy subjects, there is a need to work with different models, such as systemic inflammation and elderly subjects, that simulate applications in clinical practice. Third, the wounds were not evaluated macroscopically. The healing stages of the wounds could be visualized. However, we could not perform this because we did not have a device with a lens that could take good images. Finally, as a limitation of the study, although this study revealed the effects of growth factors at the gene expression level, examination of protein levels by western blot or ELISA could have increased the power of the study. 

## 5. Conclusions

This study revealed that the external application of a 10 kV/m electric field for 15 min leads to optimal results in terms of wound healing. Future studies should explore the effects of EFs on growth factors in greater detail, focusing on understanding the mechanisms underlying these responses. In particular, investigating the effects of varying treatment durations and densities on healing processes is critical for optimizing EF therapy protocols.

## Figures and Tables

**Table 1:** Histopathologic score to assess wound healing.

Scoring Criteria	Score			
	0	1	2	3
Re-epithelialization	None	Partial	Complete, but thin	Complete and mature
Neovascularization	None	Up to 5 vessels/ HMF	6–10 vessels/ HMF	>10 vessels/HMF
Maturation of granulation tissue	Immature	Mild maturation	Moderate maturation	Fully matured
Inflammation	None	Scant	Moderate	Abundant
Ulcer	Wide and deep ulcers, abscesses	Wide ulcers	None or very small	None

HMF: High magnification field.

**Table 2:** Primary sequences, product sizes and accession numbers of genes.

Genes	Primary Sequence	Product Size	Accession Number
ACTB (HouseKeeping)	F: CCCGCGAGTACAACCTTCTT	481 bp	NM_031144.3
	R: AACACAGCCTGGATGGCTAC		
eNOS	F: GGTTGACCAAGGCAAACCAC	247 bp	NM_021838.2
	R: CCTAATACCACAGCCGGAGG		
VEGF	F: TTCGTCCAACTTCTGGGCTC	482 bp	NM_001287111.1
	R: GCTTTCTGCTCCCCTTCTGT		
EGF	F: CCCATTGGCAAAACCAGGTG	397 bp	XM_063281339.1
	R: TCCATCGCCAGCAAATCCTT		
FGF2	F: AAAACCTGACCCGATCCCTC	121 BP	NM_019305.2
	R: CGTGACGCAGCTCCTAAAGT		

F: Forward, R: Reverse, eNOS: Endothelial nitric oxide synthase, VEGF: Vascular endothelial growth factor, EGF: Epidermal growth factor, FGF2: Fibroblast growth factor.

## Data Availability

The data that support the findings of this study are available from the corresponding author upon reasonable request.

## References

[1] Hajhosseini B., Longaker M.T., Gurtner G.C. (2020). Pressure Injury. Ann. Surg..

[2] Kottner J., Cuddigan J., Carville K., Balzer K., Berlowitz D., Law S., Litchford M., Mitchell P., Moore Z., Pittman J. (2020). Pressure ulcer/injury classification today: An international perspective. J. Tissue Viability.

[3] Pan Y., Yang D., Zhou M., Liu Y., Pan J., Wu Y., Huang L., Li H. (2023). Advance in topical biomaterials and mechanisms for the intervention of pressure injury. iScience.

[4] Donaldson C., de Abreu M.G., Mascha E.J., Rowbottom J., Harvester E., Khanna A., Sura T., Sessler D.I., Patarroyo F.R., Gulluoglu A. (2024). Pressure injury treatment by intermittent electrical stimulation (PROTECT-2): Protocol for a multicenter randomized clinical trial. Trials.

[5] Haesler E., Cuddigan J., Carville K., Moore Z., Kottner J., Ayello E.A., Berlowitz D., Carruth A., Yee C.Y., Cox J. (2024). Protocol for the Development of the Fourth Edition of the Prevention and Treatment of Pressure Ulcers/Injuries: Clinical Practice Guideline Using GRADE Methods. Adv. Skin. Wound Care.

[6] Gould L.J., Alderden J., Aslam R., Barbul A., Bogie K.M. (2024). WHS guidelines for the treatment of pressure ulcers-2023 update. Wound Repair Regen..

[7] Jeong J.W., Lee S., Park J.H. (2024). Closed-incision negative pressure wound therapy (NPWT) in elderly patients following sacral pressure sore reconstruction. BMC Geriatr..

[8] Shi J., Gao Y., Tian J., Li J., Xu J., Mei F., Li Z. (2023). Negative pressure wound therapy for treating pressure ulcers. Cochrane Database Syst. Rev..

[9] Srivastava G.K., Martinez-Rodriguez S., Md Fadilah N.I., Looi Qi Hao D., Markey G., Shukla P. (2024). Progress in Wound-Healing Products Based on Natural Compounds, Stem Cells, and MicroRNA-Based Biopolymers in the European, USA, and Asian Markets: Opportunities, Barriers, and Regulatory Issues. Polymers.

[10] Westby M.J., Dumville J.C., Soares M.O., Stubbs N., Norman G. (2017). Dressings and topical agents for treating pressure ulcers. Cochrane Database Syst. Rev..

[11] Wilkinson H.N., Hardman M.J. (2020). Wound healing: Cellular mechanisms and pathological outcomes. Open Biol..

[12] Werner S., Grose R. (2003). Regulation of wound healing by growth factors and cytokines. Physiol. Rev..

[13] Liang Y., Tian H., Liu J., Lv Y., Wang Y., Zhang J., Huang Y. (2020). Application of stable continuous external electric field promotes wound healing in pig wound model. Bioelectrochemistry.

[14] Yang J., Liu X., Wang W., Chen Y., Liu J., Zhang Z., Wu C., Jiang X., Liang Y., Zhang J. (2022). Bioelectric fields coordinate wound contraction and re-epithelialization process to accelerate wound healing via promoting myofibroblast transformation. Bioelectrochemistry.

[15] Ji R., Teng M., Zhang Z., Wang W., Zhang Q., Lv Y., Zhang J., Jiang X. (2020). Electric field down-regulates CD9 to promote keratinocytes migration through AMPK pathway. Int. J. Med. Sci..

[16] Wang W., Huang W., Liu J., Zhang Z., Ji R., Wu C., Zhang J., Jiang X. (2023). Electric field promotes dermal fibroblast transdifferentiation through activation of RhoA/ROCK1 pathway. Int. J. Med. Sci..

[17] Rajendran S.B., Challen K., Wright K.L., Hardy J.G. (2021). Electrical Stimulation to Enhance Wound Healing. J. Funct. Biomater..

[18] Qin X., Shen H., Chen R., Ye R., Hu C. (2024). Reliability of evidence supporting the role of electrical stimulation in the treatment of pressure ulcers. Int. Wound J..

[19] Szołtys-Brzezowska B., Bańkowska A., Piejko L., Zarzeczny R., Nawrat-Szołtysik A., Kloth L.C., Polak A. (2023). Electrical Stimulation in the Treatment of Pressure Injuries: A Systematic Review of Clinical Trials. Adv. Skin. Wound Care.

[20] Wei X., Guan L., Fan P., Liu X., Liu R., Liu Y., Bai H. (2020). Direct Current Electric Field Stimulates Nitric Oxide Production and Promotes NO-Dependent Angiogenesis: Involvement of the PI3K/Akt Signaling Pathway. J. Vasc. Res..

[21] Chen Y., Ye L., Guan L., Fan P., Liu R., Liu H., Chen J., Zhu Y., Wei X., Liu Y. (2018). Physiological electric field works via the VEGF receptor to stimulate neovessel formation of vascular endothelial cells in a 3D environment. Biol. Open.

[22] Luo R., Dai J., Zhang J., Li Z. (2021). Accelerated Skin Wound Healing by Electrical Stimulation. Adv. Healthc. Mater..

[23] Stadler I., Zhang R.Y., Oskoui P., Whittaker M.S., Lanzafame R.J. (2004). Development of a simple, noninvasive, clinically relevant model of pressure ulcers in the mouse. J. Investig. Surg..

[24] Yudhantoro L., Hidajat N.N., Ismiarto Y.D., Ismono D. (2019). Histopathological Effects of Ageratum Leaf Extract (*Ageratum conyzoides*) on Wound Healing Acceleration After Acute Excisional Wound on Epidermis in Type 2 Diabetes Mellitus Model of Sprague Dawley Rats (*Rattus norvegicus*). Maj. Kedokt. Bdg..

[25] Katoh K. (2023). Effects of Electrical Stimulation of the Cell: Wound Healing, Cell Proliferation, Apoptosis, and Signal Transduction. Med. Sci..

[26] Farber P.L., Isoldi F.C., Ferreira L.M. (2021). Electric Factors in Wound Healing. Adv. Wound Care.

[27] Sari Y., Sutrisna E. (2019). The effect of short duration of electrical stimulation on wound healing in acute wound in a rat model. Wound Med..

[28] Raziyeva K., Kim Y., Zharkinbekov Z., Kassymbek K., Jimi S., Saparov A. (2021). Immunology of Acute and Chronic Wound Healing. Biomolecules.

[29] Shin S.H., Koh Y.G., Lee W.G., Seok J., Park K.Y. (2023). The use of epidermal growth factor in dermatological practice. Int. Wound J..

[30] Duda D.G., Fukumura D., Jain R.K. (2004). Role of eNOS in neovascularization: NO for endothelial progenitor cells. Trends Mol. Med..

[31] Chen Z., Haus J.M., Chen L., Wu S.C., Urao N., Koh T.J., Minshall R.D. (2020). CCL28-induced CCR10/eNOS interaction in angiogenesis and skin wound healing. Faseb J.

[32] Arora M., Harvey L.A., Glinsky J.V., Nier L., Lavrencic L., Kifley A., Cameron I.D. (2020). Electrical stimulation for treating pressure ulcers. Cochrane Database Syst. Rev..

